# Serum Na-Cl value from routine blood tests reflects CO_2_ retention in amyotrophic lateral sclerosis

**DOI:** 10.1371/journal.pone.0358772

**Published:** 2026-09-18

**Authors:** Isamu Yamakawa, Ryota Tamura, Hiroyuki Yabata, Takahito Tsukamoto, Shuhei Kobashi, Yoshitaka Tamaki, Nobuhiro Ogawa, Akihiro Kitamura, Makoto Urushitani

**Affiliations:** Department of Neurology, Shiga University of Medical Science, Otsu, Shiga, Japan; International University of Health and Welfare, School of Medicine, JAPAN

## Abstract

**Background:**

Respiratory failure is the leading cause of death in amyotrophic lateral sclerosis (ALS). Early detection of CO_2_ retention is crucial for assessing respiratory failure severity and guiding noninvasive positive pressure ventilation (NPPV) management. However, arterial blood gas analysis is invasive and uncomfortable for routine monitoring.

**Objective:**

We investigated whether the serum sodium-chloride difference (Na-Cl value), calculated from routine blood tests, could serve as a screening marker for CO_2_ retention in ALS patients.

**Methods:**

This retrospective study included 88 ALS patients with 116 paired samples of arterial blood gas and serum electrolyte data. We analyzed correlations between Na-Cl value and blood gas parameters (HCO_3_^−^, PCO_2_), performed receiver operating characteristic (ROC) analysis for detecting CO_2_ retention (PCO_2_ ≥ 45 mmHg), and examined relationships with respiratory function (%FVC).

**Results:**

Na-Cl value showed strong correlation with HCO_3_^−^ (r = 0.78, p < 0.001) and PCO_2_ (r = 0.71, p < 0.001). Na-Cl ≥ 37 mEq/L demonstrated sensitivity of 85.11% and specificity of 69.57% for detecting PCO_2_ ≥ 45 mmHg, with negative predictive value of 87.27% (AUC = 0.842). Na-Cl ≥ 39 mEq/L achieved specificity of 92.75%. Patients with %FVC < 50% had significantly higher Na-Cl, PCO_2_, and HCO_3_^−^ values.

**Conclusions:**

Serum Na-Cl value serves as a simple, noninvasive screening marker for CO_2_ retention in ALS. Na-Cl ≥ 37 mEq/L warrants blood gas analysis, while Na-Cl ≥ 39 mEq/L provides specificity >90%. This marker can be calculated from routine blood tests without additional cost, making it suitable for frequent monitoring and reducing the risk of missing intervention timing.

## Introduction

Respiratory failure is the leading cause of death in amyotrophic lateral sclerosis (ALS), occurring in nearly all patients during the natural course of disease progression. Progressive weakness of the diaphragm and intercostal muscles inevitably leads to restrictive ventilatory impairment, and the majority of patients develop chronic progressive CO_2_ retention [1–3]. Critically, hypercapnia often develops insidiously before the emergence of overt respiratory symptoms, making early detection essential [4]. Timely initiation of noninvasive positive pressure ventilation (NPPV) upon detection of respiratory insufficiency has been demonstrated to prolong survival and improve quality of life in ALS patients [5–7].

Arterial blood gas (ABG) analysis remains the gold standard for detecting CO_2_ retention and holds particular importance in ALS monitoring, as its results are not influenced by bulbar involvement and require no active patient collaboration [8]. However, the procedure’s inherently invasive nature and associated patient discomfort severely limit its feasibility for frequent, routine assessment in outpatient clinical practice. While pulmonary function tests such as forced vital capacity and maximal inspiratory pressure are commonly used to monitor respiratory function and serve as reliable indicators of CO_2_ retention risk in patients without significant bulbar dysfunction, these parameters may show limited utility in predicting hypercapnia in patients with prominent bulbar symptoms who have difficulty forming a tight lip seal around the spirometry tube, thereby preventing accurate measurement [9,10]. Transcutaneous CO_2_ monitoring and overnight oximetry provide noninvasive alternatives but require specialized equipment and are not routinely available in all clinical settings [11]. Therefore, a simple, noninvasive, and readily accessible screening marker derived from routine laboratory tests would be of substantial clinical value.

In chronic respiratory acidosis due to CO_2_ retention, compensatory metabolic alkalosis develops through renal mechanisms: the kidneys enhance bicarbonate (HCO_3_^−^) reabsorption while increasing chloride (Cl^−^) excretion to maintain acid-base homeostasis [12,13]. This physiological adaptation results in an elevated serum sodium-chloride difference (Na-Cl value), which can be readily calculated from routine serum chemistry panels. The Na-Cl value has theoretical advantages over isolated chloride measurements, as it accounts for variations in sodium levels and better reflects the degree of metabolic compensation [14,15]. Indeed, in patients with chronic obstructive pulmonary disease (COPD), Alfaro et al. demonstrated that chronic hypercapnia leads to an increase in the strong ion difference (the difference between strong cations [Na + , K + , Ca2+] and strong anions [Cl^−^, lactate]). This increase in the strong ion difference is primarily due to a decrease in plasma chloride concentration and reflects metabolic compensation for respiratory acidosis [16]. Although previous studies have reported associations between serum chloride levels and both survival and timing of NPPV initiation in ALS patients [17], the specific utility of the Na-Cl value as a quantitative screening marker for CO_2_ retention has not been systematically evaluated.

The objectives of this study were: (i) to investigate the correlation between serum Na-Cl value and PaCO_2_ in ALS patients; (ii) to establish clinically applicable cutoff values for screening CO_2_ retention; and (iii) to examine the relationship between Na-Cl value and respiratory function parameters (%FVC) to evaluate its complementary role in respiratory monitoring. We hypothesized that the Na-Cl value would serve as a useful surrogate marker for CO_2_ retention and could facilitate earlier detection of hypercapnia in routine clinical practice.

## Methods

### Study design and patients

This retrospective observational study included patients with ALS who visited the Department of Neurology at Shiga University of Medical Science between July 1, 2016, and October 31, 2025. A total of 107 patients with ALS who underwent both arterial blood gas analysis and routine blood chemistry examination were enrolled in this study, yielding 165 paired samples of blood gas and serum electrolyte data.

### Exclusion criteria

To minimize confounding factors that could affect acid-base balance, we excluded patients with the following conditions: (1) Hypokalemia (serum potassium <3.5 mEq/L), which can induce metabolic alkalosis [18]; (2) use of supplemental oxygen at the time of blood sampling; and (3) continuous use of non-invasive positive pressure ventilation (NPPV) throughout the day. Initially, 107 patients with 165 blood samples were identified. After applying the exclusion criteria, 88 patients with 116 samples remained for analysis. Among these 116 samples, 74 had concurrent measurements of %FVC, allowing for analysis of the relationships between respiratory function and blood gas parameters (Na-Cl, HCO_3_^−^, and PCO_2_).

### Laboratory measurements

Arterial blood gas analysis was performed to determine pH, PCO_2_, partial pressure of oxygen (PO_2_), HCO_3_^−^, and base excess. Serum electrolytes including sodium (Na), chloride (Cl), and potassium (K) were measured using standard automated analyzers. The Na-Cl value was calculated by subtracting the chloride concentration from the sodium concentration. Respiratory function was assessed by measuring forced vital capacity (FVC) and expressing it as percent predicted forced vital capacity (%FVC).

### Statistical analysis

Continuous variables are presented as mean ± standard deviation or median (range) as appropriate. Categorical variables are expressed as numbers and percentages. Spearman’s correlation coefficients were used to assess relationships between Na-Cl and blood gas parameters (HCO_3_^−^, PCO_2_) as well as respiratory function (%FVC). Comparisons between two groups (CO_2_ retention defined as PCO_2_ ≥ 45 mmHg vs. PCO_2_ < 45 mmHg; respiratory dysfunction as %FVC < 50% vs. %FVC ≥ 50%) were performed using the Mann-Whitney U test.

Receiver operating characteristic (ROC) curve analysis was conducted to evaluate the diagnostic performance of Na-Cl for detecting CO_2_ retention. The area under the curve (AUC), optimal cutoff value (determined by Youden’s index), sensitivity, specificity, positive predictive value (PPV), and negative predictive value (NPV) were calculated. Multiple cutoff values (37, 38, 39, and 40 mEq/L) were examined to assess the sensitivity-specificity trade-off. Because multiple samples were obtained from some patients, we performed a sensitivity analysis using only the first sample from each patient (n = 88) to confirm that the results were not influenced by repeated measurements from the same patient. All statistical analyses were performed with EZR (Jichi Medical University, Tochigi, Japan), which is a graphical user interface for R (The R Foundation for Statistical Computing, Vienna, Austria). More precisely, it is a modified version of R commander designed to add statistical functions frequently used in biostatistics [19]. Statistical significance was set at p < 0.05 (two-tailed).

### Standard protocol approvals, registrations, and patient consents

This retrospective study was approved by the Ethics Committee of Shiga University of Medical Science (approval number: R2025-092, approved on November 28, 2025). Medical records and laboratory data were accessed for research purposes from December 28, 2025. During data collection, the authors had access to information that could identify individual participants. Written informed consent was waived by the ethics committee due to the retrospective nature of the study, and an opt-out approach was used to ensure participants’ rights to refuse participation.

## Results

A total of 88 ALS patients were enrolled in this study, yielding 116 serum samples for analysis. Detailed patient characteristics are presented in Table 1.

**Table 1:** Patient characteristics.

Characteristic	Value
Number of patients	88
Number of samples	116
**Sex, n (%)**	
Male	47 (53.4%)
Female	41 (46.6%)
Age, years	67.1 ± 10.0
Median (range)	68 (34–88)
**Site of onset, n (%)**	
Spinal	55 (62.5%)
Bulbar	31 (35.2%)
Respiratory	1 (1.1%)
PLS	1 (1.1%)
Disease duration, years	2.7 ± 2.4
Median (range)	2.0 (0.2–12.4)
ALSFRS-R score	31.3 ± 9.2
Median (range)	32.0 (3–46)

ALSFRS-R: Revised Amyotrophic Lateral Sclerosis Functional Rating Scale; PLS: Primary Lateral Sclerosis.

**Fig 1:**
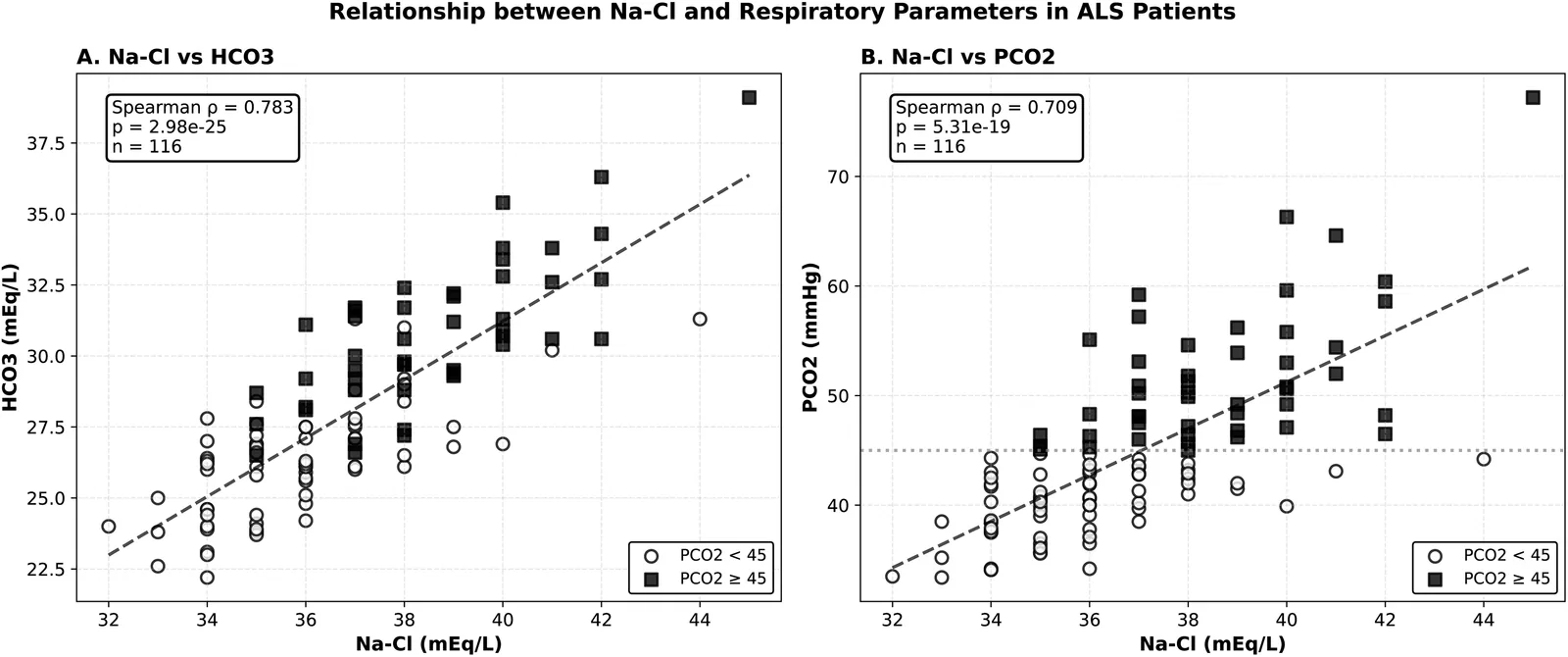
Correlation analysis between Na-Cl value and blood gas parameters. Scatter plots showing the relationships between Na-Cl and (A) HCO_3_^−^ (r = 0.78, p < 0.001) and (B) PCO_2_ (r = 0.71, p < 0.001) in 116 samples from 88 ALS patients. The regression lines (red) demonstrate strong positive correlations. Statistical comparisons were performed using the Spearman correlation coefficient.

Correlation analysis of the 116 samples revealed a strong positive correlation between Na-Cl and HCO_3_^−^ (Pearson r = 0.78, p < 0.001), and a moderate to strong positive correlation between Na-Cl and PCO_2_ (Spearman’s r = 0.71, p < 0.001) (Fig 1).

The threshold of 45 mmHg for PCO_2_ was established based on the EFNS guidelines [20] and the Canadian Thoracic Society guideline [21]. These guidelines recommend considering the initiation of NPPV when PCO_2_ exceeds 45 mmHg. The threshold of %FVC < 50% was based on the AAN practice parameter [5]. Jimenez et al. [22] also used %FVC < 50% predicted and PCO_2_ > 45 mmHg as criteria for NPPV in their cohort study.

**Fig 2:**
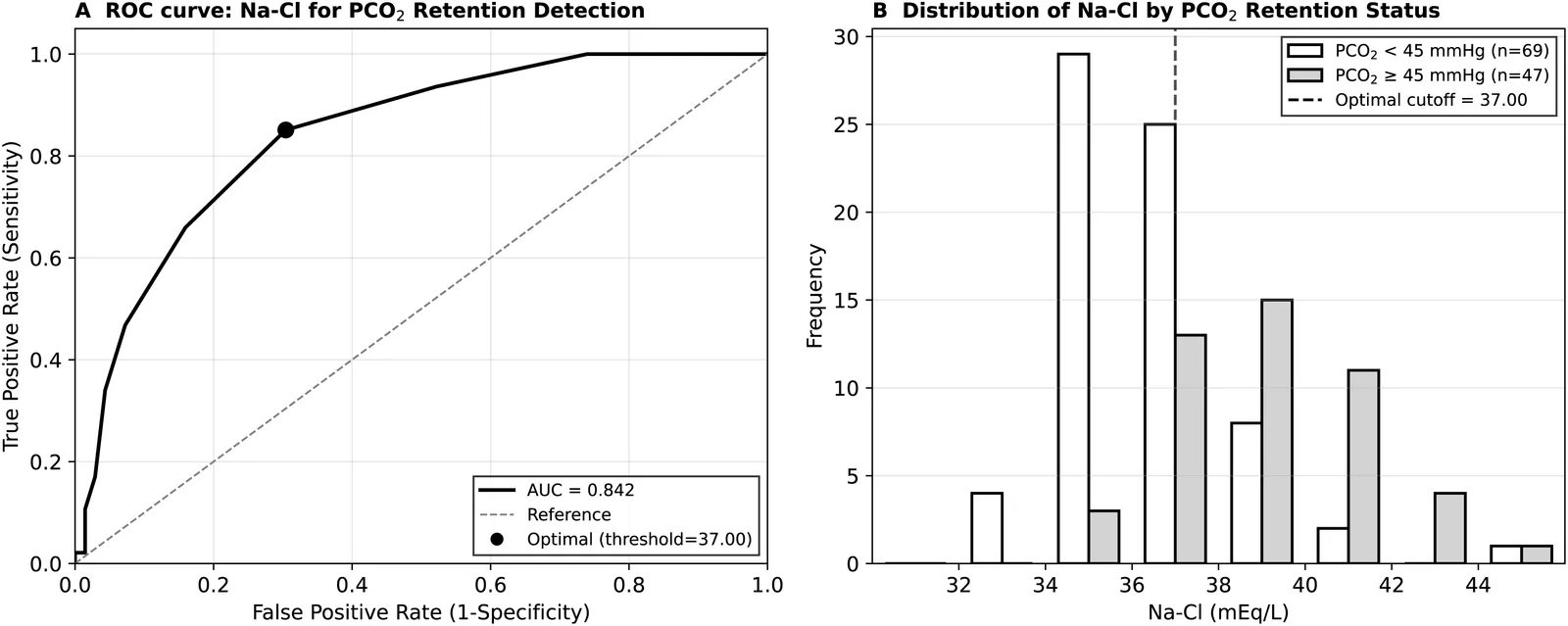
ROC curve analysis for detection of CO_2_ retention. (A) Receiver operating characteristic curve showing the diagnostic performance of Na-Cl for detecting CO_2_ retention (PCO_2_ ≥ 45 mmHg). The optimal cutoff value of 37 mEq/L (black dot) yielded an AUC of 0.842 with sensitivity 85.11% and specificity 69.57%. (B) Distribution of Na-Cl values stratified by CO_2_ retention status, showing the optimal cutoff at 37 mEq/L (dashed line).

Regarding the detection of CO_2_ retention (defined as PCO_2_ ≥ 45 mmHg), receiver operating characteristic (ROC) curve analysis was performed. Using a cutoff value of Na-Cl ≥ 37 mEq/L, the analysis demonstrated favorable diagnostic performance with a sensitivity of 85.11%, specificity of 69.57%, positive predictive value (PPV) of 65.57%, negative predictive value (NPV) of 87.27%, and area under the curve (AUC) of 0.842 (Fig 2).

For applications prioritizing high specificity to minimize false positives, a cutoff value of 39 mEq/L achieved a specificity of 92.75% (exceeding the 90% threshold), with a sensitivity of 46.81%, PPV of 81.47%, and NPV of 71.91%. This cutoff resulted in only 5 false positives (5/69 non-CO_2_-retention patients).

Stratification by CO_2_ retention status revealed that patients with PCO_2_ ≥ 45 mmHg demonstrated significantly higher values compared to those with PCO_2_ < 45 mmHg for Na-Cl (38.60 ± 2.15 vs. 35.84 ± 1.99 mEq/L, p < 0.001) and HCO_3_^−^ (30.74 ± 2.58 vs. 26.29 ± 1.96 mEq/L, p < 0.001) (Table 2).

**Table 2:** Comparison of laboratory parameters by CO_2_ retention status.

Group	n	Na-Cl (mEq/L)	HCO_3_^−^ (mEq/L)
All samples	116	36.96 ± 2.46	28.09 ± 3.12
PCO_2_ ≥ 45 mmHg	47	**38.60 ± 2.15**	**30.74 ± 2.58**
PCO_2_ < 45 mmHg	69	35.84 ± 1.99	26.29 ± 1.96
*p value*	–	<0.001	<0.001

Data are presented as mean ± standard deviation. CO_2_ retention was defined as PCO_2_ ≥ 45 mmHg. p values were calculated using the Mann-Whitney U test.

Comparison of blood gas parameters by ALS onset type revealed no significant differences. Na-Cl values in sample from spine-onset patients (36.91 ± 2.61 mEq/L, n = 74) were nearly identical to those from bulbar-onset patients (36.95 ± 2.06 mEq/L, n = 39, p = 0.75; S1A Fig). Similarly, neither HCO_3_^−^ (p = 0.65; S1B Fig) nor PCO_2_ (p = 0.34; S1C Fig) differed between groups, indicating that Na-Cl value reflects respiratory status independent of disease onset site. This comparison was restricted to spinal-onset and bulbar-onset samples, because the respiratory-onset and PLS cases together comprised only three samples.

**Fig 3:**
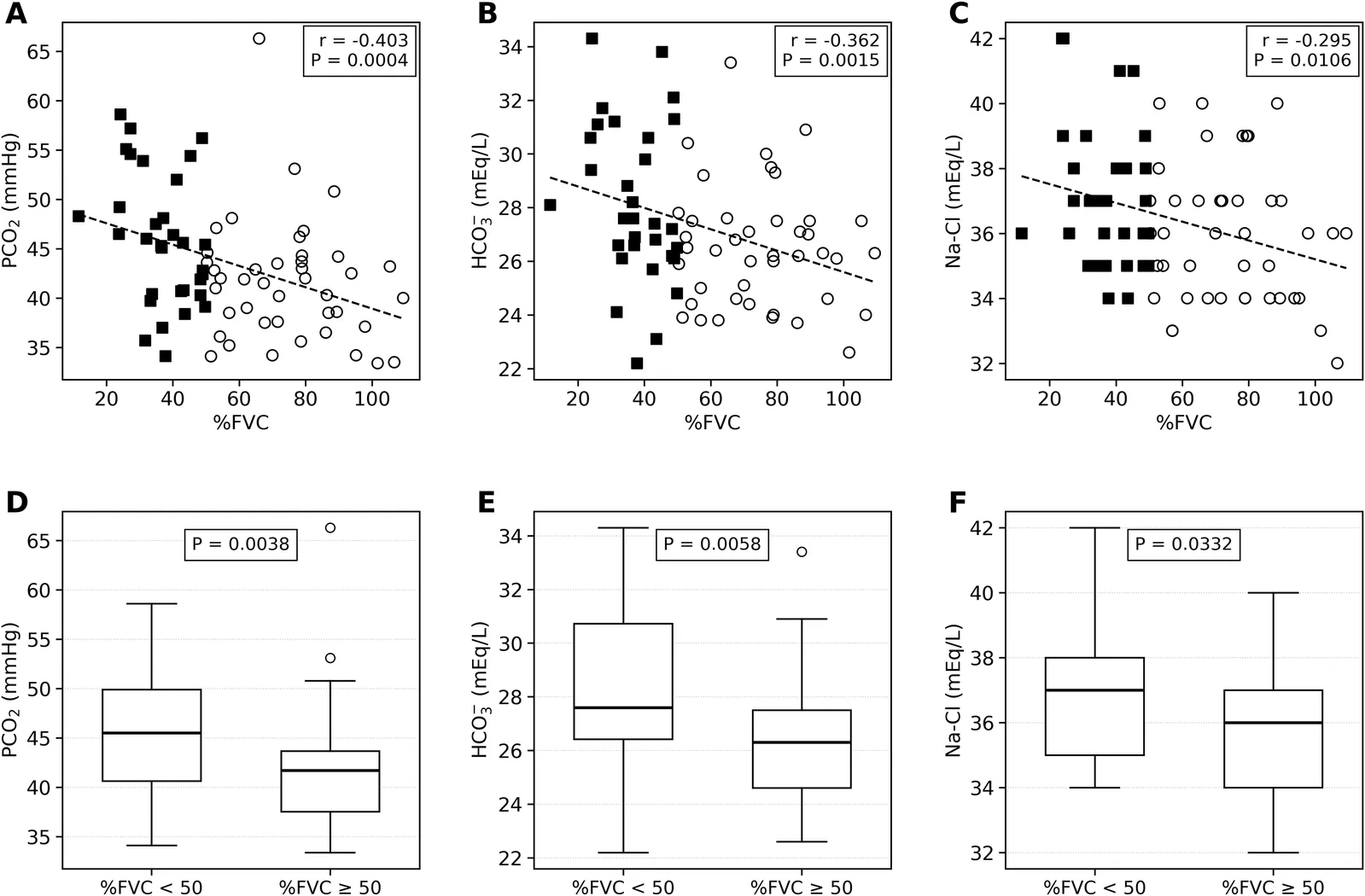
Relationship between respiratory function and blood gas parameters. (A–C) Black squares represent patients with CO2 retention (PCO2 ≥ 45 mmHg), and open circles represent patients without CO2 retention (PCO2 < 45 mmHg). Scatter plots showing negative correlations between %FVC and PCO_2_ (r=−0.403, p = 0.0004), HCO_3_^−^ (r=−0.362, p = 0.0015), and Na-Cl (r=−0.295, p = 0.0106) in the 74 samples with concurrent %FVC measurements. (D–F) Box plots comparing the same parameters between patients with %FVC < 50% (respiratory dysfunction) and %FVC ≥ 50% (preserved respiratory function). Patients with %FVC < 50% had significantly higher values for all three parameters (p < 0.05 for all comparisons). Statistical comparisons were performed using the Spearman correlation coefficient (A–C) and the Mann-Whitney U test (D–F).

Furthermore, we examined the relationship between respiratory function (%FVC) and laboratory parameters in the 74 samples with concurrent %FVC measurements. Significant negative correlations were observed between %FVC and PCO_2_ (r=−0.403, p = 0.0004), HCO_3_^−^ (r=−0.362, p = 0.0015), and Na-Cl (r=−0.295, p = 0.0106). Patients stratified by respiratory function showed that those with %FVC < 50% demonstrated significantly higher values compared to those with %FVC ≥ 50% for Na-Cl (p = 0.0332), PCO_2_ (p = 0.0038) and HCO_3_^−^ (p = 0.0058) (Fig 3).

As a sensitivity analysis, we repeated the primary analysis using only the first sample from each patient (n = 88). The correlations were comparable or even stronger. The correlation between Na-Cl and HCO_3_^−^ was Spearman’s r = 0.802 (vs. r = 0.783 in the full dataset), and the correlation between Na-Cl and PCO_2_ was r = 0.736 (vs. r = 0.709 in the full dataset). In the ROC analysis, the AUC improved to 0.868, with comparable diagnostic performance at both cutoff values: for Na-Cl ≥ 37, sensitivity was 88.2%, specificity 68.5%, and negative predictive value (NPV) 90.2%; for Na-Cl ≥ 39, sensitivity was 52.9% and specificity 94.4%. These results indicate that the inclusion of repeated measurements did not affect our conclusions (S1 Table).

## Discussion

This study demonstrated that serum Na-Cl value serves as a simple and useful screening marker for CO_2_ retention in patients with ALS. Our analysis of 116 samples from 88 ALS patients revealed strong correlations between Na-Cl and both HCO_3_^−^ (r = 0.78) and PCO_2_ (r = 0.71). Most importantly, Na-Cl ≥ 37 mEq/L showed excellent screening performance for detecting CO_2_ retention (PCO_2_ ≥ 45 mmHg) with high sensitivity (85.11%) and a notably high negative predictive value (87.27%). This finding has substantial clinical implications, as Na-Cl can be easily and repeatedly measured from routine serum chemistry panels without the need for invasive arterial blood gas sampling, making it particularly valuable for regular monitoring of respiratory status in ALS patients during outpatient follow-up.

We propose a stepwise approach using different Na-Cl cutoff values depending on the clinical context. For screening purposes, Na-Cl ≥ 37 mEq/L can be used to broadly detect potential CO_2_ retention with 85% sensitivity. However, when a more definitive diagnosis is required, Na-Cl ≥ 39 mEq/L provides higher diagnostic accuracy with specificity of 92.75% and positive predictive value of 81.47%. This stepwise approach enables clinicians to appropriately stratify patients who require confirmatory arterial blood gas analysis.

One of the important advantages of using Na-Cl value is that it can be elevated even when individual Na or Cl values remain within the normal range. This allows for the detection of CO_2_ retention that might otherwise be overlooked in routine electrolyte assessments, thereby reducing the risk of missing clinically significant respiratory compromise in daily practice. Furthermore, our study demonstrated significant negative correlations between respiratory function (%FVC) and Na-Cl, PCO_2_, and HCO_3_^−^, with patients having %FVC < 50% showing significantly higher values for all three parameters. This suggests that Na-Cl value may reflect the degree of respiratory function decline. Importantly, Na-Cl values were independent of ALS onset type (spine vs. bulbar), suggesting that this marker reflects the degree of respiratory muscle weakness rather than the site of disease initiation. This universality across onset types enhances the clinical utility of Na-Cl value as a screening tool, as it requires no adjustment for patient subgroups and can be applied uniformly across the ALS population.

Direct measurement of serum bicarbonate or total CO_2_ would likely serve as a more direct indicator of metabolic compensation. However, in many countries, including Japan, venous bicarbonate is not routinely measured as part of standard blood chemistry panels. In contrast, serum Na and Cl are universally measured. Therefore, the Na-Cl value can be calculated without any additional testing.

The strong correlation between Na-Cl and PCO_2_ observed in our ALS cohort can be attributed to the pathophysiological characteristics of the disease. ALS primarily causes pure restrictive ventilatory impairment due to progressive respiratory muscle weakness, leading to CO_2_ retention with compensatory metabolic alkalosis, which is reflected in the elevated Na-Cl value. Importantly, comorbidities that elevate the anion gap (AG) are less common in ALS patients during disease progression, particularly before requiring full-time NPPV or oxygen therapy. This allows the Na-Cl value to serve as a reliable surrogate marker for respiratory status in this population. However, caution is needed when interpreting Na-Cl values in other clinical contexts. In conditions causing metabolic alkalosis such as hypokalemia [18], or in diseases with elevated anion gap (such as diabetic ketoacidosis, lactic acidosis, or renal failure), Na-Cl values may be elevated independent of CO_2_ retention [23]. Therefore, these confounding conditions should be carefully excluded when an elevated Na-Cl value is observed, and the clinical context must always be taken into consideration when using Na-Cl as a screening marker for respiratory compromise.

Previous studies have examined the relationship between serum chloride levels and respiratory status in ALS. Manera et al. [17] reported that serum chloride levels were associated with survival and the timing of NPPV initiation. However, their study focused on chloride alone as a prognostic indicator. In contrast, our study evaluated the Na-Cl difference as a screening marker for CO_2_ retention. The Na-Cl difference has the advantage of accounting for fluctuations in sodium levels, thereby more accurately reflecting metabolic compensation. Furthermore, our study presented specific cutoff values accompanied by diagnostic performance data, enabling direct clinical application as a screening tool. Regarding COPD, Alfaro et al. [16] used a physicochemical approach to analyze acid-base changes in patients with chronic hypercapnia. They demonstrated that the strong ion difference (SID; i.e., [Na⁺ + K⁺ + Ca²⁺] − [Cl^−^ + lactate]) increases with CO_2_ retention, primarily due to a decrease in plasma chloride. Our study applied a simpler approach using Na-Cl, a value that can be easily calculated from routine blood tests. Furthermore, the ALS population differs from the COPD population in that it presents with pure restrictive ventilatory dysfunction without an obstructive component. This difference may make the relationship between Na-Cl and CO_2_ retention more straightforward.

Several potential confounding factors warrant consideration. In this cohort, renal function was generally preserved (mean serum creatinine 0.537 ± 0.200 mg/dL, BUN 15.7 ± 5.1 mg/dL, eGFR 118.9 ± 64.9 mL/min/1.73 m²). Only 2 of the 116 specimens had creatinine levels exceeding 1.0 mg/dL, and no patient showed elevated BUN. In ALS, serum creatinine is typically low owing to muscle atrophy, which leads to overestimation of eGFR. In the 30 cases in which cystatin C was measured, the cystatin C–based eGFR was 67.0 ± 18.5 mL/min/1.73 m², and no patient showed severe renal impairment. Only 7 of the 88 patients (8.0%) were receiving diuretics or agents with natriuretic effects, and excluding these patients did not alter the results (AUC = 0.834). It should be noted that the Na-Cl value may not accurately reflect CO_2_ retention in the presence of conditions that independently affect electrolyte balance. Such conditions include metabolic alkalosis (e.g., vomiting, diuretic use), high-anion-gap metabolic acidosis (e.g., diabetic ketoacidosis, lactic acidosis, renal failure), and dehydration. Clinicians should take these conditions into account when interpreting Na-Cl values in clinical practice.

This study has several limitations. First, it was a single-center retrospective study, which may introduce selection bias. Second, the relatively small sample size warrants validation in larger prospective studies. Future multicenter collaborative studies are needed to confirm the generalizability of these findings. Third, some patients provided multiple samples, which were treated as independent observations in the primary analysis. However, a sensitivity analysis using only the first sample from each patient confirmed that the results were robust. Fourth, potential confounding factors such as diuretic use and gastrointestinal symptoms were assessed retrospectively rather than through systematic prospective evaluation. Nevertheless, these factors were unlikely to have significantly affected the results, given the low prevalence of diuretic use (8.0%), the absence of documented gastrointestinal symptoms, and the exclusion of patients with hypokalemia. Fifth, this study did not include a validation cohort; future multicenter prospective studies with an independent validation cohort are therefore needed.

In conclusion, this study demonstrates that the Na-Cl value, which can be easily calculated from routine serum chemistry panels, has the potential to reduce the risk of overlooking CO_2_ retention in ALS patients. Importantly, this holds true even when individual Na and Cl values are within their respective normal ranges. Based on our findings, we recommend that when Na-Cl ≥ 37 mEq/L is observed, clinicians should maintain a high index of suspicion for CO_2_ retention (PCO_2_ ≥ 45 mmHg) and consider performing arterial blood gas analysis for further evaluation. When Na-Cl ≥ 39 mEq/L is detected, the likelihood of significant CO_2_ retention is substantially elevated, warranting prompt investigation with blood gas analysis. Implementation of this simple screening approach in routine clinical practice may help prevent delayed detection of respiratory compromise and enable timely intervention in ALS patients, ultimately contributing to improved respiratory management and patient outcomes.

## Supporting information

S1 Fig Comparison of Na-Cl, HCO_3_^−^ and PCO_2_ values by ALS onset type (spine vs bulbar). Box plots showing the distribution of (A) Na-Cl, (B) HCO_3_^−^, and (C) PCO_2_ values stratified by ALS onset type. Samples from spine-onset patients (n = 74) are shown on the left, and samples from bulbar-onset patients (n = 39) on the right for each parameter. Statistical comparisons were performed using the Mann-Whitney U test. No significant differences were observed between the two groups for any parameter: Na-Cl (spine: 36.91 ± 2.61 mEq/L vs. bulbar: 36.95 ± 2.06 mEq/L, p = 0.75), HCO_3_^−^ (p = 0.65), and PCO_2_ (spine: 44.75 ± 7.03 mmHg vs. bulbar: 44.27 ± 7.24 mmHg, p = 0.34). These findings indicate that Na-Cl values reflect respiratory status independent of disease onset site.
(TIF)

S1 Table Sensitivity analysis: comparison of results using all samples (n = 116) versus the first sample per patient (n = 88). (DOCX)

S1 Data Data anonymized. (XLSX)
