# Supplementary material for: Serum Na-Cl value from routine blood tests reflects CO2 retention in amyotrophic lateral sclerosis
Source: PLoS One. 2026 Sep 18;21(9):e0358772. doi: 10.1371/journal.pone.0358772 (PMC13588376; doi:10.1371/journal.pone.0358772)
Supplement: Supplementary file 1 [file pone.0358772.s002.docx]

**S1 Table. Sensitivity analysis: comparison of results using all samples (n = 116) versus the first sample per patient (n = 88).**

|  | **All samples (n = 116)** | **First sample only (n = 88)** |
| --- | --- | --- |
| **Correlation analysis (Spearman r)** |  |  |
| Na-Cl vs. HCO₃⁻ | 0.783 (p < 0.001) | 0.802 (p < 0.001) |
| Na-Cl vs. PCO₂ | 0.709 (p < 0.001) | 0.736 (p < 0.001) |
| **ROC analysis for CO₂ retention (PCO₂ ≥45 mmHg)** |  |  |
| AUC | 0.842 | 0.868 |
| **Cutoff Na-Cl ≥37 mEq/L** |  |  |
| Sensitivity (%) | 85.1 | 88.2 |
| Specificity (%) | 69.6 | 68.5 |
| PPV (%) | 65.6 | 63.8 |
| NPV (%) | 87.3 | 90.2 |
| **Cutoff Na-Cl ≥39 mEq/L** |  |  |
| Sensitivity (%) | 46.8 | 52.9 |
| Specificity (%) | 92.8 | 94.4 |
| PPV (%) | 81.5 | 85.7 |
| NPV (%) | 71.9 | 73.0 |

*AUC, area under the curve; PPV, positive predictive value; NPV, negative predictive value. CO₂ retention was defined as PCO₂ ≥45 mmHg. The sensitivity analysis used only the first sample from each patient to eliminate potential bias from repeated measurements.*
