# Supplementary material for: Serum Na-Cl value from routine blood tests reflects CO2 retention in amyotrophic lateral sclerosis
Source: PLoS One. 2026 Sep 18;21(9):e0358772. doi: 10.1371/journal.pone.0358772 (PMC13588376; doi:10.1371/journal.pone.0358772)
Supplement: Peer review history

# Peer review: Decision Letter 0

**DOI:** 10.1371/journal.pone.0358772.r001

**Authors:** Tatsuo Shimosawa

12 Aug 2026

Dear Dr. Yamakawa,

Please submit your revised manuscript by Sep 26 2026 11:59PM. If you will need more time than this to complete your revisions, please reply to this message or contact the journal office at plosone@plos.org. When you're ready to submit your revision, log on to https://www.editorialmanager.com/pone/ and select the 'Submissions Needing Revision' folder to locate your manuscript file.

- A letter that responds to each point raised by the academic editor and reviewer(s). You should upload this letter as a separate file labeled 'Response to Reviewers'.
- A marked-up copy of your manuscript that highlights changes made to the original version. You should upload this as a separate file labeled 'Revised Manuscript with Track Changes'.
- An unmarked version of your revised paper without tracked changes. You should upload this as a separate file labeled 'Manuscript'.

If you would like to make changes to your financial disclosure, please include your updated statement in your cover letter. Guidelines for resubmitting your figure files are available below the reviewer comments at the end of this letter.

If applicable, we recommend that you deposit your laboratory protocols in protocols.io to enhance the reproducibility of your results. Protocols.io assigns your protocol its own identifier (DOI) so that it can be cited independently in the future. For instructions see: https://journals.plos.org/plosone/s/submission-guidelines#loc-laboratory-protocols. Additionally, PLOS ONE offers an option for publishing peer-reviewed Lab Protocol articles, which describe protocols hosted on protocols.io. Read more information on sharing protocols at https://plos.org/protocols?utm_medium=editorial-email&utm_source=authorletters&utm_campaign=protocols.

As the corresponding author, your ORCID iD is verified in the submission system and will appear in the published article. PLOS supports the use of ORCID, and we encourage all coauthors to register for an ORCID iD and use it as well. Please encourage your coauthors to verify their ORCID iD within the submission system before final acceptance, as unverified ORCID iDs will not appear in the published article. *Only*  the individual author can complete the verification step; PLOS staff *cannot*  verify ORCID iDs on behalf of authors.

We look forward to receiving your revised manuscript.

Kind regards,

Tatsuo Shimosawa, M.D., Ph.D.

Academic Editor

PLOS One

Journal Requirements:

When submitting your revision, we need you to address these additional requirements.

1. Please ensure that your manuscript meets PLOS ONE's style requirements, including those for file naming. The PLOS ONE style templates can be found at

https://journals.plos.org/plosone/s/file?id=wjVg/PLOSOne_formatting_sample_main_body.pdf and

https://journals.plos.org/plosone/s/file?id=ba62/PLOSOne_formatting_sample_title_authors_affiliations.pdf

2. Thank you for stating the following financial disclosure:

“Intramural Research Grant from Shiga University of Medical Science.”

Please state what role the funders took in the study.  If the funders had no role, please state: "The funders had no role in study design, data collection and analysis, decision to publish, or preparation of the manuscript.” If this statement is not correct you must amend it as needed.

Please include this amended Role of Funder statement in your cover letter; we will change the online submission form on your behalf.

3. We note that your Data Availability Statement is currently as follows:

“All relevant data are within the manuscript and its Supporting Information files.”

Please confirm at this time whether or not your submission contains all raw data required to replicate the results of your study. Authors must share the “minimal data set” for their submission. PLOS defines the minimal data set to consist of the data required to replicate all study findings reported in the article, as well as related metadata and methods (https://journals.plos.org/plosone/s/data-availability#loc-minimal-data-set-definition).

For example, authors should submit the following data:

- The values behind the means, standard deviations and other measures reported;

- The values used to build graphs;

- The points extracted from images for analysis.

Authors do not need to submit their entire data set if only a portion of the data was used in the reported study.

If your submission does not contain these data, please either upload them as Supporting Information files or deposit them to a stable, public repository and provide us with the relevant URLs, DOIs, or accession numbers. For a list of recommended repositories, please see https://journals.plos.org/plosone/s/recommended-repositories.

If there are ethical or legal restrictions on sharing a de-identified data set, please explain them in detail (e.g., data contain potentially sensitive information, data are owned by a third-party organization, etc.) and who has imposed them (e.g., an ethics committee). Please also provide contact information for a data access committee, ethics committee, or other institutional body to which data requests may be sent. If data are owned by a third party, please indicate how others may request data access.

4. Please ensure that you refer to Figures 1 and 3 in your text as, if accepted, production will need this reference to link the reader to the figure.

5. We notice that your supplementary figures are uploaded with the file type 'Figure'. Please amend the file type to 'Supporting Information'. Please ensure that each Supporting Information file has a legend listed in the manuscript after the references list.

6. If the reviewer comments include a recommendation to cite specific previously published works, please review and evaluate these publications to determine whether they are relevant and should be cited. There is no requirement to cite these works unless the editor has indicated otherwise.

**Additional Editor Comments:**

Three experts raised several concerns, specially data analysis based upon proper statistical analysis is requested.

[Note: HTML markup is below. Please do not edit.]

Reviewers' comments:

Reviewer's Responses to Questions

**Comments to the Author**

1. Is the manuscript technically sound, and do the data support the conclusions?

Reviewer #1: Yes

Reviewer #2: Yes

Reviewer #3: Yes

**********

2. Has the statistical analysis been performed appropriately and rigorously?

Reviewer #1: Yes

Reviewer #2: Yes

Reviewer #3: No

**********

3. Have the authors made all data underlying the findings in their manuscript fully available?

The PLOS Data policy

Reviewer #1: Yes

Reviewer #2: Yes

Reviewer #3: Yes

**********

4. Is the manuscript presented in an intelligible fashion and written in standard English?

Reviewer #1: Yes

Reviewer #2: Yes

Reviewer #3: Yes

**********

Reviewer #1: This is a small single center clinical observation study, reporting that in ALS patients the Na Cl concentration difference is indicative of the degree of Co 2 retention in ALS patients.

The charm of this simple correlative study with real world data is that it establishes an link between routine blood parameters (serum electrolytes) and a clinically relevant readout (Co2 retention levels). This is potentially very useful because Co2 retention is a result of chronic hypoventilation and one of the most important survival factors in ALS.

The research question very sound and overall well presented. The data analysis is straightforward and nicely presented.

Given that the approach is so simple, it is not clear to me why they did not address the biggest weakness of the study (which they freely admit): the fact that this was done only in a single cohort. The impact of the study would be much strengthened if they provided a second (validation) cohort. As its stands now this is an, albeit intriguing, pilot study.

Also, in the results section (page 7, line 134) crucial information (the p value) is missing.

Reviewer #2: This manuscript addresses a clinically relevant and practical issue and is generally well organized. However, several important issues require clarification. Please address the following comments.

Major comments

1. The study included 116 samples obtained from 88 patients. Please clarify how multiple samples from the same patient were handled in the statistical analyses. If all samples were treated as independent observations, this should be acknowledged and discussed as a limitation of the study.

2. The rationale for recommending both 37 and 39 mEq/L as clinically useful cutoff values should be clarified. Although the optimal cutoff was determined using Youden's index, the rationale for recommending an additional cutoff of 39 mEq/L should be explained more explicitly. Although this cutoff provides high specificity (>90%), the basis for selecting 39 mEq/L and proposing a stepwise approach is not sufficiently described.

Minor comments

1 Given the broad readership of this journal, please briefly explain the clinical significance of the selected thresholds (PaCO₂ ≥45 mmHg and %VC <50%).

2. On page 7, line 134, the p-value appears to be incomplete or missing. Please verify and revise the relevant text as appropriate.

3. In Fig. 3A–C, the meanings of the black squares and open circles are not explained in the figure legend. Please clarify what these symbols represent.

Reviewer #3: This study demonstrated that the serum Na–Cl difference is associated with CO2 retention and respiratory function, both of which correlate with disease severity in patients with amyotrophic lateral sclerosis (ALS).. These findings have important clinical implications. However, there are several points to consider.

1. In Some facilities, serum bicarbonate concentration, or total CO2, may be measured directly, which may be a more immediate marker of the disease severity. However, it is necessary to note that such measurement of bicarbonate concentration is not a routine practice in Japan, and that Na–Cl is a more stable marker.

2. Some patients had multiple data. Such patients with multiple measurements have greater effects on the results studied. It is necessary to apply a mixed-effects model to account for within-patient correlation, or conduct a sensitivity analysis using only the first sample of each patient with repeated measurements.

3. It is necessary to examine other factors that affect serum chloride or bicarbonate levels, such as renal function, diuretics use, diarrhea, or vomiting. In particular, the authors should mention diuretic use and the indices of renal function (e.g., serum creatinine and/or urea levels).

4. Several studies demonstrated an association between serum chloride and CO2 accumulation or respiratory function. Authors are required to discuss the additive value or differences of this study over those studies.

5. The cutoff values, such as CO2 levels of 45 mmHg or %VC of 50%, require their validities. The basics or evidences for using these values must be provided.

**********

what does this mean?). If published, this will include your full peer review and any attached files.

If you choose “no”, your identity will remain anonymous but your review may still be made public.

**Do you want your identity to be public for this peer review?**  For information about this choice, including consent withdrawal, please see our Privacy Policy

Reviewer #1: No

Reviewer #2: No

Reviewer #3: No

**********

[NOTE: If reviewer comments were submitted as an attachment file, they will be attached to this email and accessible via the submission site. Please log into your account, locate the manuscript record, and check for the action link "View Attachments". If this link does not appear, there are no attachment files.]

To ensure your figures meet our technical requirements, please review our figure guidelines: https://journals.plos.org/plosone/s/figures

You may also use PLOS’s free figure tool, NAAS, to help you prepare publication quality figures: https://journals.plos.org/plosone/s/figures#loc-tools-for-figure-preparation.

NAAS will assess whether your figures meet our technical requirements by comparing each figure against our figure specifications.

# Author comment: Author response to Decision Letter 1

**DOI:** 10.1371/journal.pone.0358772.r002

19 Aug 2026

Response to Reviewers

Manuscript: PONE-D-26-02384

"Serum Na-Cl value from routine blood tests reflects CO₂ retention in amyotrophic lateral sclerosis"

We would like to thank the Academic Editor and the three reviewers for their helpful comments and suggestions. We have carefully revised the manuscript to address each point. Below, we provide our responses to each comment. The reviewer comments are shown in bold.

Journal Requirements

1. PLOS ONE style requirements

We have checked and revised the manuscript to follow PLOS ONE’s style requirements, including file naming.

2. Financial disclosure — Role of funder

Please state what role the funders took in the study.

We have added the following statement to the cover letter: "The funders had no role in study design, data collection and analysis, decision to publish, or preparation of the manuscript."

3. Data Availability Statement

Please confirm whether your submission contains all raw data required to replicate the results.

We confirm that all data needed to replicate the study findings are included in the manuscript and its Supporting Information files. We have uploaded the de-identified dataset as a Supporting Information file (S1 Data).

4. Reference to Figures 1 and 3 in text

Please ensure that you refer to Figures 1 and 3 in your text.

We have confirmed that Figures 1 and 3 are cited in the Results section of the revised manuscript.

5. Supporting Information file type

Please amend the file type to ‘Supporting Information.’

We have changed the file type of the supplementary figures from ‘Figure’ to ‘Supporting Information.’ Each Supporting Information file now has a legend listed after the references.

6. Recommended citations

If the reviewer comments include a recommendation to cite specific previously published works, please review and evaluate.

No specific citations were recommended by the reviewers. However, we have added several relevant references in response to specific comments, as described below.

Additional Editor Comments

"Three experts raised several concerns, specially data analysis based upon proper statistical analysis is requested."

We thank the Editor for this important comment. To address the concerns about statistical analysis, we (1) performed a sensitivity analysis using only the first sample per patient to confirm the robustness of the results, (2) examined potential confounders including renal function and diuretic use, and (3) corrected the missing p-value. Details are provided in the responses to each reviewer below.

In addition, since the international guidelines (AAN, EFNS) use %FVC (forced vital capacity) rather than %VC (slow vital capacity) as the criterion for NIV initiation, we re-analyzed the respiratory function data using %FVC to match the guideline criteria. One patient was excluded from this analysis because the forced expiratory maneuver could not be performed adequately (the test result was recorded as a reference value only), resulting in 74 patients for the %FVC analysis (compared with 75 for the original %VC analysis). The results using %FVC showed the same trend as the original %VC analysis, with similar or stronger correlations. The revised manuscript now reports %FVC instead of %VC throughout.

Reviewer #1

Comment 1: Validation cohort

"Given that the approach is so simple, it is not clear to me why they did not address the biggest weakness of the study (which they freely admit): the fact that this was done only in a single cohort. The impact of the study would be much strengthened if they provided a second (validation) cohort."

We thank the reviewer for this suggestion. We agree that a validation cohort would strengthen the study. However, as this is a single-center study, we do not have access to an independent dataset with paired arterial blood gas and serum electrolyte data at this time. Obtaining such data from another institution would require new ethics approvals and data sharing agreements, which is difficult to complete within the revision period.

We have added the need for multicenter validation as a future direction in the Limitations section.

Comment 2: Missing p-value (page 7, line 134)

"Also, in the results section (page 7, line 134) crucial information (the p value) is missing."

We apologize for this error. The p-value has been added. The corrected text now reads: “...a moderate to strong positive correlation between Na-Cl and PCO₂ (Pearson r = 0.71, p < 0.001).”

Reviewer #2

Major Comment 1: Multiple samples from same patient

"The study included 116 samples obtained from 88 patients. Please clarify how multiple samples from the same patient were handled in the statistical analyses."

We thank the reviewer for raising this point. In the original analysis, all 116 samples were treated as independent observations. To address this concern, we performed a sensitivity analysis using only the first sample from each patient (n = 88), which eliminates any potential influence of repeated measurements.

The results of this sensitivity analysis showed that the correlations were similar or stronger compared with the original analysis: Spearman r = 0.802 for Na-Cl vs. HCO₃⁻ (vs. r = 0.783 in the full dataset) and r = 0.736 for Na-Cl vs. PCO₂ (vs. r = 0.709). The ROC analysis showed an improved AUC of 0.868 (vs. 0.842), and the diagnostic performance was similar at both cutoff values (Na-Cl ≥ 37: sensitivity 88.2%, specificity 68.5%, NPV 90.2%; Na-Cl ≥ 39: sensitivity 52.9%, specificity 94.4%).

These results confirm that the inclusion of repeated measurements did not affect our conclusions. We have added these results and a discussion of this issue to the revised manuscript. The detailed results of the sensitivity analysis are shown in S2 Table.

Major Comment 2: Rationale for both 37 and 39 mEq/L cutoffs

"The rationale for recommending both 37 and 39 mEq/L as clinically useful cutoff values should be clarified."

We have clarified the rationale in the revised manuscript. The cutoff of 37 mEq/L was determined by Youden’s index from the ROC analysis as the optimal threshold, with the best balance of sensitivity (85.1%) and specificity (69.6%). This cutoff is intended for screening, where the goal is to avoid missing CO₂ retention (NPV = 87.3%).

The cutoff of 39 mEq/L was selected because it provides specificity above 90% (92.8%), with only 5 false positives among 69 non-CO₂-retention samples. This higher cutoff is useful when confirmatory testing is limited or when avoiding unnecessary arterial blood gas analysis is preferred.

This stepwise approach — screening at ≥ 37 mEq/L and stronger suspicion at ≥ 39 mEq/L — allows clinicians to use different thresholds depending on the clinical situation. We have revised the Discussion to explain this more clearly.

Minor Comment 1: Clinical significance of PaCO₂ ≥ 45 mmHg and %VC < 50%

"Given the broad readership of this journal, please briefly explain the clinical significance of the selected thresholds."

We have added explanations in the revised manuscript. PaCO₂ ≥ 45 mmHg was selected based on established international guidelines. The EFNS guidelines (Andersen et al., Eur J Neurol 2012) and the Canadian Thoracic Society guideline (McKim et al., Can Respir J 2011) recommend considering non-invasive ventilation (NIV) when PaCO₂ exceeds 45 mmHg, because this indicates clinically significant CO₂ retention.

%FVC < 50% was based on the AAN practice parameter (Miller et al., Neurology 2009), which recommends considering NIV when FVC falls below 50% predicted. This cutoff has been supported by recent studies including Jimenez et al. (Muscle Nerve 2024). In addition, PEG placement is generally recommended when %FVC is still above 50%. Therefore, these thresholds are important clinical decision points in ALS management. The references have been added to the revised manuscript.

Since these guidelines use %FVC (forced vital capacity) as the criterion, we re-analyzed the respiratory function data using %FVC instead of %VC (slow vital capacity). One patient was excluded because the forced expiratory maneuver could not be performed adequately, resulting in 74 patients for the %FVC analysis. The results were similar or stronger than the original analysis, and the revised manuscript now reports %FVC throughout.

Minor Comment 2: Missing p-value (page 7, line 134)

"On page 7, line 134, the p-value appears to be incomplete or missing."

We have corrected this. Please see our response to Reviewer #1, Comment 2.

Minor Comment 3: Figure 3A–C symbol legend

"In Fig. 3A–C, the meanings of the black squares and open circles are not explained in the figure legend."

We have updated the figure legend for Figure 3. Black squares represent patients with CO₂ retention (PCO₂ ≥ 45 mmHg), and open circles represent patients without CO₂ retention (PCO₂ < 45 mmHg). This is the same notation used in Figure 1.

Reviewer #3

Comment 1: Bicarbonate measurement vs. Na-Cl

"In some facilities, serum bicarbonate concentration, or total CO₂, may be measured directly, which may be a more immediate marker of the disease severity. However, it is necessary to note that such measurement of bicarbonate concentration is not a routine practice in Japan, and that Na-Cl is a more stable marker."

We agree with the reviewer and have added this discussion in the revised manuscript. Direct measurement of serum bicarbonate would be a more immediate marker. However, as the reviewer correctly points out, venous bicarbonate is not routinely measured in standard blood chemistry panels in Japan and many other countries. Serum Na and Cl, on the other hand, are universally measured, so the Na-Cl value can be calculated without any additional testing. Also, Na-Cl is less affected by sample handling conditions (such as air exposure during transport) compared with direct bicarbonate measurement. We have added these points to the Discussion.

Comment 2: Mixed-effects model for repeated measures

"Some patients had multiple data. It is necessary to apply a mixed-effects model to account for within-patient correlation, or conduct a sensitivity analysis using only the first sample of each patient with repeated measurements."

We thank the reviewer for this recommendation. As the reviewer suggested, we performed a sensitivity analysis using only the first sample from each patient (n = 88) to eliminate any potential influence of repeated measurements.

The results showed that the correlations were similar or stronger compared with the full dataset: Spearman r = 0.802 for Na-Cl vs. HCO₃⁻ (vs. r = 0.783 in the full dataset) and r = 0.736 for Na-Cl vs. PCO₂ (vs. r = 0.709). The ROC AUC improved from 0.842 to 0.868, and the diagnostic performance at both cutoff values was similar.

These results confirm that the repeated measurements did not affect our findings. The sensitivity analysis results have been added to the revised manuscript.

Comment 3: Confounding factors (renal function, diuretics, diarrhea, vomiting)

"It is necessary to examine other factors that affect serum chloride or bicarbonate levels, such as renal function, diuretics use, diarrhea, or vomiting."

We thank the reviewer for this suggestion. We reviewed the clinical records and performed additional analyses. The results are as follows:

Renal function:

Serum creatinine and BUN data were available for all 116 samples. The mean serum creatinine was 0.537 ± 0.200 mg/dL (range 0.16–1.37), BUN was 15.7 ± 5.1 mg/dL (range 6.3–31.7), and creatinine-based eGFR was 118.9 ± 64.9 mL/min/1.73m². Only 2 of 116 samples (1.7%) had creatinine > 1.0 mg/dL, and no patient showed elevated BUN. These results indicate that clinically significant renal impairment was uncommon in this cohort.

Diuretic use:

Among the 88 patients, only 7 (8.0%) were receiving diuretics or medications with natriuretic properties at the time of blood sampling (thiazides: n = 3; loop diuretics: n = 2; SGLT2 inhibitor: n = 1; ARNI [sacubitril/valsartan]: n = 3; some patients received multiple agents). The mean Na-Cl in patients receiving these medications (37.88 ± 1.64 mEq/L) was slightly higher than in those without (36.89 ± 2.50 mEq/L). However, when we excluded all samples from these patients (n = 108 samples), the correlations were almost the same (Na-Cl vs. HCO₃⁻: Spearman r = 0.782, p < 0.001; Na-Cl vs. PCO₂: r = 0.704, p < 0.001; AUC = 0.834). This shows that these medications did not confound the results.

Gastrointestinal factors:

It should be noted that Na-Cl values may not accurately reflect CO₂ retention in the presence of conditions that independently affect electrolyte balance, such as metabolic alkalosis (e.g., vomiting, diuretic use), metabolic acidosis with elevated anion gap (e.g., diabetic ketoacidosis, lactic acidosis, renal failure), and dehydration. Although episodes of diarrhea or vomiting were not systematically recorded in our study, we excluded patients with hypokalemia (< 3.5 mEq/L), which often accompanies significant vomiting, which would have removed many cases with significant vomiting. We recommend that clinicians consider these conditions when interpreting Na-Cl values in clinical practice.

Comment 4: Comparison with existing studies on chloride and CO₂

"Several studies demonstrated an association between serum chloride and CO₂ accumulation or respiratory function. Authors are required to discuss the additive value or differences of this study over those studies."

We agree and have expanded the Discussion. The main differences from previous studies are:

Previous studies have examined the relationship between serum chloride and respiratory status in ALS. Manera et al. (2023) reported that serum chloride levels are associated with survival and timing of NPPV initiation. However, their study focused on isolated chloride as a prognostic marker, while our study evaluated the Na-Cl difference as a screening marker for CO₂ retention. The Na-Cl difference has the advantage of accounting for changes in sodium levels and better reflecting metabolic compensation.

In addition, our study provided specific cutoff values with diagnostic performance data (sensitivity, specificity, PPV, NPV) for detecting CO₂ retention, which allows direct clinical application as a screening tool.

Regarding COPD, Alfaro et al. (1996) used a physicochemical approach to analyze acid-base changes in patients with chronic hypercapnia, and showed that the strong ion difference (the difference between strong cations [Na⁺, K⁺, Ca²⁺] and strong anions [Cl⁻, lactate]) increased with CO₂ retention, mainly due to a decrease in plasma chloride. Our study applied a simpler approach using Na-Cl, which can be easily calculated from routine blood tests. The ALS population also differs from COPD in that it presents with pure restrictive ventilatory impairment without the obstructive component, which may make the relationship between Na-Cl and CO₂ retention more straightforward.

These comparisons have been added to the Discussion.

Comment 5: Validity of cutoff values (PCO₂ 45 mmHg, %VC 50%)

"The cutoff values, such as CO₂ levels of 45 mmHg or %VC of 50%, require their validities. The basics or evidences for using these values must be provided."

We have added the evidence for these cutoff values in the revised manuscript:

PCO₂ ≥ 45 mmHg: This threshold is based on the EFNS guidelines (Andersen et al., Eur J Neurol 2012) and the Canadian Thoracic Society guideline (McKim et al., Can Respir J 2011), which recommend considering NIV when PCO₂ exceeds 45 mmHg. This value is the upper limit of normal and indicates clinically significant CO₂ retention.

%FVC < 50%: This threshold is based on the AAN practice parameter (Miller et al., Neurology 2009), which recommends considering NIV when FVC falls below 50% predicted. Recent studies, including Jimenez et al. (Muscle Nerve 2024), have also supported this cutoff. In addition, PEG placement is generally recommended when %FVC is still above 50%, making this an important decision point in ALS management.

In addition, since these guidelines use %FVC (forced vital capacity) as the criterion, we re-analyzed the respiratory function data using %FVC instead of %VC (slow vital capacity) to match the guideline criteria. One patient was excluded because the forced expiratory maneuver could not be performed adequately, resulting in 74 patients for the %FVC analysis. The results were similar or stronger than the original %VC analysis, confirming the validity of these cutoff values.

These references have been added to the revised manuscript.

During the revision process, we recalculated the patient characteristics in Table 1 from the raw data and corrected minor errors. Because each sample was obtained at a different time point, we recalculated the values based on all 116 samples (at the time of blood sampling) rather than per patient. The corrected values are as follows: Age (67.1 ± 10.0 years; Median 68), Disease duration (2.7 ± 2.4 years; Median 2.0; range 0.2–12.4), and ALSFRS-R score (31.3 ± 9.2; Median 32.0; range 3–46). These corrections do not affect any of the study conclusions. The corrected values can be verified from the de-identified dataset uploaded as S1 Data.

We hope that these revisions address the concerns raised. We are happy to make any further changes if needed.

Attachment Submitted filename: Response_to_Reviewers.docx

# Peer review: Decision Letter 1

**DOI:** 10.1371/journal.pone.0358772.r003

**Authors:** Tatsuo Shimosawa

7 Sep 2026

<p>Serum Na-Cl value from routine blood tests reflects CO2 retention in amyotrophic lateral sclerosis

PONE-D-26-02384R1

Dear Dr. Yamakawa,

We’re pleased to inform you that your manuscript has been judged scientifically suitable for publication and will be formally accepted for publication once it meets all outstanding technical requirements.

Within one week, you’ll receive an e-mail detailing the required amendments. When these have been addressed, you’ll receive a formal acceptance letter and your manuscript will be scheduled for publication.

An invoice will be generated when your article is formally accepted. Please note, if your institution has a publishing partnership with PLOS and your article meets the relevant criteria, all or part of your publication costs will be covered. Please make sure your user information is up-to-date by logging into Editorial Manager at Editorial Manager® and clicking the ‘Update My Information' link at the top of the page. For questions related to billing, please contact billing support.

If your institution or institutions have a press office, please notify them about your upcoming paper to help maximize its impact. If they’ll be preparing press materials, please inform our press team as soon as possible -- no later than 48 hours after receiving the formal acceptance. Your manuscript will remain under strict press embargo until 2 pm Eastern Time on the date of publication. For more information, please contact onepress@plos.org.

Kind regards,

Tatsuo Shimosawa, M.D., Ph.D.

Academic Editor

PLOS One

Additional Editor Comments (optional):

Reviewers' comments:

Reviewer's Responses to Questions

**Comments to the Author**

Reviewer #1: All comments have been addressed

Reviewer #2: All comments have been addressed

Reviewer #3: All comments have been addressed

**********

2. Is the manuscript technically sound, and do the data support the conclusions?

Reviewer #1: Yes

Reviewer #2: Yes

Reviewer #3: Yes

**********

3. Has the statistical analysis been performed appropriately and rigorously?

Reviewer #1: Yes

Reviewer #2: Yes

Reviewer #3: Yes

**********

4. Have the authors made all data underlying the findings in their manuscript fully available?

The PLOS Data policy

Reviewer #1: Yes

Reviewer #2: Yes

Reviewer #3: Yes

**********

5. Is the manuscript presented in an intelligible fashion and written in standard English?

Reviewer #1: Yes

Reviewer #2: Yes

Reviewer #3: Yes

**********

Reviewer #1: My concerns have been adequately addressed and the manuscript is now improved so that it is suitable for publication from my view.

Reviewer #2: Thank you for your thoughtful revision. The authors have adequately addressed my previous concerns, and the additional analyses have provided further support for the findings. I have no further comments.

Reviewer #3: All comments raised on the previous version have been properly addressed in this revision by authors.

**********

what does this mean?). If published, this will include your full peer review and any attached files.

If you choose “no”, your identity will remain anonymous but your review may still be made public.

**Do you want your identity to be public for this peer review?**  For information about this choice, including consent withdrawal, please see our Privacy Policy

Reviewer #1: No

Reviewer #2: No

Reviewer #3: **Yes:** Norio Hanafusa

**********

# Editor report: Acceptance letter

**DOI:** 10.1371/journal.pone.0358772.r004

**Authors:** Tatsuo Shimosawa

PONE-D-26-02384R1

PLOS One

Dear Dr. Yamakawa,

I'm pleased to inform you that your manuscript has been deemed suitable for publication in PLOS One. Congratulations! Your manuscript is now being handed over to our production team.

At this stage, our production department will prepare your paper for publication. This includes ensuring the following:

* All references, tables, and figures are properly cited

* All relevant supporting information is included in the manuscript submission,

* There are no issues that prevent the paper from being properly typeset

You will receive further instructions from the production team, including instructions on how to review your proof when it is ready. Please keep in mind that we are working through a large volume of accepted articles, so please give us a few days to review your paper and let you know the next and final steps.

Lastly, if your institution or institutions have a press office, please let them know about your upcoming paper now to help maximize its impact. If they'll be preparing press materials, please inform our press team within the next 48 hours. Your manuscript will remain under strict press embargo until 2 pm Eastern Time on the date of publication. For more information, please contact onepress@plos.org.

You will receive an invoice from PLOS for your publication fee after your manuscript has reached the completed accept phase. If you receive an email requesting payment before acceptance or for any other service, this may be a phishing scheme. Learn how to identify phishing emails and protect your accounts at https://explore.plos.org/phishing.

If we can help with anything else, please email us at customercare@plos.org.

Thank you for submitting your work to PLOS One and supporting open access.

Kind regards,

PLOS One Editorial Office Staff

on behalf of

Prof. Tatsuo Shimosawa

Academic Editor

PLOS One
